# What is the meaning and nature of active play for today's children in the UK?

**DOI:** 10.1186/1479-5868-8-15

**Published:** 2011-03-07

**Authors:** Rowan Brockman, Kenneth R Fox, Russell Jago

**Affiliations:** 1 Centre for Exercise, Nutrition & Health Sciences, School for Policy Studies, University of Bristol, Bristol, UK

## Abstract

**Background:**

Preventing the decline in physical activity which occurs around 10-11 years of age is a public health priority. Physically active play can make unique contributions to children's development which cannot be obtained from more structured forms of physical activity. Encouraging active play in children's leisure time has potential to increase physical activity levels while promoting optimal child development. Aspired wisdom states that contemporary British children no longer play outdoors, but systematic evidence for this is lacking. We need to build a more informed picture of contemporary children's play before we consider interventions to increase it.

**Methods:**

Eleven focus groups were conducted with 77, 10-11 year old children from four primary schools in Bristol, UK. Focus groups examined: 1) children's perceptions of 'play'; 2) how much of their play is active play; and 3) contexts of children's active play. All focus groups were audio-taped and transcribed verbatim. Data were analysed using a thematic approach.

**Results:**

Children's perceptions of play were broad and included both physically active and sedentary behaviours. Children reported that they frequently engaged in active play and valued both the physical and social benefits it provided. Whereas boys frequently reported having a 'kick about' or riding bikes as their preferred forms of active play, girls were less likely to report a specific activity. Additionally, boys reported greater independent mobility in their active play compared to girls. Finally, boys were more likely to report playing with neighbourhood friends but girls more frequently reported playing with family members.

**Conclusions:**

Promoting active play in children's leisure time may increase the physical activity of children, but interventions may need to be tailored according to gender.

## Background

Regular physical activity in children is associated with lower body mass [1], blood pressure [2], insulin levels [3] and improved mental wellbeing [4,5]. Despite its health benefits, many children and young people do not meet the current UK guidelines of an hour per day of moderate to vigorous physical activity (MVPA) on most days of the week [6]. Moreover, physical activity levels decline during childhood, with the end of primary school (10-11 years) being a critical stage of change [7,8]. Preventing the decline in physical activity that occurs at this age is therefore a key public health target [9].

Physical activity in children takes place in a number of contexts, including sports clubs, adult-organised activities during and after school, active travel and informal play. The determinants of physical activity are likely to be different for each context [10]. Most children obtain physical activity from more than one context. This may be desirable as different types of activity provide different health and social benefits [11]. The majority of the data regarding children's physical activity is limited to sports clubs, adult-organised activities during and after school and active travel [12]. A relatively neglected area of research is physical activity obtained through informal play [13].

The benefits of play are wide reaching and extend beyond the health gains from physical activity. Play has been widely acknowledged as an essential part of human development [14-16] and is recognised by the UN High Commission for Human Rights as a basic right of every child [17]. Although there is a lack of agreement amongst academics on an overarching definition of 'play', common characteristics of play behaviours are that they are freely chosen, personally directed, intrinsically motivated, spontaneous and pleasurable [18,19]. There are many different types of play, which vary according to age and setting [20]. During the primary school years, children are reported to engage in a vigorous form of play termed 'physical activity play' [13], or 'active play'.

Active play may involve symbolic activity or games with rules; the activity may be social or solitary, but the distinguishing features are a playful context, combined with activity that is significantly above resting metabolic rate [21]. Active play tends to occur sporadically, with frequent rest periods [22], which makes it difficult to record. However, recent research has found active play to be associated with moderate to vigorous physical activity, particularly during the after school period, in a sample of UK 10-11 year olds [23]. Additionally, active play may make important cognitive, physical, social and emotional contributions to children's development which are not necessarily obtained from more structured forms of physical activity [24]. These may include developing creativity, resolving conflicts in peer groups, social interaction skills, conquering fears and building resilience to face future challenges.

Whilst acknowledging that some forms of indoor play could be perceived as physically active, including active games consoles, 'soft play' centres or active board games, for the purposes of this study, we defined active play as "unstructured physical activity which takes place outdoors in a child's free time" [25]. A characteristic of outdoor active play in comparison to indoor active play, is that it often takes place in the absence of parents, providing opportunities for children to 'make it on their own' and develop a sense of independence [14]. This is particularly important during the transition from primary to secondary school (10-11 years), when parents naturally begin to afford their children increased licence to be independently physically active [26,27]. Thus, encouraging independent, outdoor active play in children's free time may be an exceptional way to increase physical activity levels while promoting optimal child development in this age group.

Despite its broad developmental and health benefits, contemporary British children are reported to spend less time engaging in active play compared with previous generations. A study of UK children's play in 1973 found that 75% of children aged 15 years and under were observed playing outdoors near their homes, mainly on roads and the pavement [28]. In contrast, data collected for the 2005 National Travel Survey suggested that only 15% of children aged 5-15 years played outside near their homes [29]. A decreased sense of community in neighbourhoods, parents' and children's concerns about safety and a middle-class culture of 'over-scheduling' children have all been cited as possible influences [14,30].

There are few recent and systematic studies in the UK that have described the nature of children's play [24]. For example, we know very little about how children in the UK today perceive play; the extent to which it involves physical activity; what children actually do during their active play; where children actively play, and who children actively play with. Moreover, research has rarely given children themselves the opportunity to discuss their play behaviours [31]. Understanding more about children's perspectives of play provides the critical first step in designing future interventions to increase active play. As there is limited available evidence in this area, we employed qualitative methods to address the following research questions among a sample of 10-11 year old children in the UK:

1) What are 10-11 year old children's perceptions of 'play'?

2) How much of this is 'active play'?

3) What are the contexts of children's active play?

i) What do they do in their active play?

ii) Where do they engage in active play?

iii) With whom do they engage in active play?

## Methods

A total of 77, 10-11 year old children were recruited from four primary schools in Bristol, UK. The schools were recruited to represent the socio-economic diversity of the local area based on the Index of Multiple Deprivation (IMD). The IMD is a UK Government-produced measure of deprivation that includes assessments of income, employment, health and education [32]. The IMD was obtained for the postcode of each school and thus represented a measure of deprivation for the school and not the individual participant. Based on the IMDs for the postcodes of all schools within a 10 mile radius of the University of Bristol, one school was recruited from the lowest quartile (Low SES school), one from the lower-middle quartile (low/middle SES school), one from the upper-middle SES quartile (middle/high SES school) and one from the highest SES quartile (High SES school). The study was approved by the School of Applied Community and Health Studies Ethics Committee at the University of Bristol (ref 016/09) and informed parental consent and child assent were obtained for all participants [33]. The researcher who carried out data collection in schools had enhanced Criminal Records Bureau (CRB) clearance.

A 'recruitment' session was held for all Year 6 pupils (10-11 years of age) at each school where children were invited to participate in a research study about physical activity and play. Focus groups were chosen as the method of data collection. Focus groups are an effective method of collecting qualitative data from children as the thoughts and ideas of other members of the group often help participants verbalise their responses in a comfortable, safe and supportive environment [34,35]. Depending on the number of consenting participants, two or three focus groups were held at each school, with a range of 4-8 children in each group. Each focus group lasted 30-40 minutes, was conducted by the first author and was digitally recorded. All focus groups took place in the winter of 2009. The focus groups had a semi-structured design with follow-up probes on key topics of interest. Questions were developed by the first author and piloted in a school before being finalised.

The focus groups questions explored how children perceive 'play' ("when you hear the word 'play', what do you think of?") and contexts of active play (e.g. "over the past week, what did you do in active play", "where did you do it" and "who did you play with?) with follow-up probes and prompting where necessary. It is important to note that for the first question regarding children's perceived meanings of 'play', children were asked for the meaning of 'play' and not 'active play'. This was to ensure responses were not biased towards outdoor, physical activity-based interpretations. Following this initial question, children were provided with the researcher's definition of active play, which was "any activity which takes place outdoors in your own free time which isn't organised by an adult." In order to limit response bias, the importance of honest, individual answers was stressed and also the fact that the focus group was not a test but an enquiry into children's perceptions. Confidentiality was also discussed.

### Analyses

All recordings were transcribed verbatim and anonymised. Tapes were erased and destroyed after transcription. All identifying data was removed from the transcripts. Transcripts will be retained in locked storage units for six years and then destroyed by shredding. Thematic analyses were conducted in two phases. First, key themes were identified by reading the transcripts line by line and marking the text with codes that described the content of the response [36]. Codes were entered as 'tree nodes' (labels that describe themes in a hierarchical format) in a newly created database in NVivo (Version 8, QSR, Southport, UK) and references were extracted from this database.

## Results

Eleven focus groups were conducted with 77 participants from 4 schools, with the sample being 64% female. There were 15 participants from the low SES school, 24 from the low/middle SES school, 15 from the middle/high SES school and 23 from the high SES school.

### Children's perceptions of 'play'

Participants were asked the question, "When you hear the word 'play', what do you think of?" with the follow-up probe, "what does 'play' mean to you?" Participants' responses to how they perceive 'play' were divided into two major themes: 1) Perceptions of active play; 2) Perceptions of non-active play.

### Perceptions of active play

Many participants, both male and female, described 'play' in terms of some form of physical activity. Generally these activities were unstructured and based on a sense of 'letting off steam.'

*"Like running round" *(Male, high SES)

*"Burning energy so we're not that like, mad in class" *(Female, low/middle SES)

*"Everyone running around and having fun" *(Female, middle/high SES)

*"Just have a kick around with a football" *(Male, low SES)

Additionally, activities were often described as taking place outdoors and in a social context:

*"When you say play I think of playing tag, outside" *(Female, low SES)

*"Racing my friends on the field" *(Male, middle/high SES)

*"Going outside with my friends" *(Male, high SES)

*"I think of play like um, playing outside with your friends and, doing new stuff, every day" *(Female, low/middle SES)

Finally, many participants equated active play with a sense of freedom from rules or structure.

*"Play just means doing what you want and running around" *(Male, low SES)

*"Running, jumping, just mucking about really" *(Female, low/middle SES)

*"Um, sort of messing around like, yeah" *(Male, low/middle SES)

*"Free" *(Female, middle/high SES)

### Perceptions of non-active play

Some participants, both male and female, perceived play in terms of sedentary activities, many of which involved the use of computers or games consoles:

*"Computer games" *(Female, high SES)

*"My sort of play's playing chess with other people online on my mum's laptop" *(Male, low SES)

*"Some like um computer games are active play because the adult doesn't organise it" *(Male, high SES)

*"You can play indoors like on video games" *(Female, low SES)

Additionally, a few participants mentioned other forms of non-active play in their perceptions:

*"Um, playing music" *(Male, high SES)

*"Board games" *(Female, middle/high SES)

*"Role playing and stuff" *(Male, low/middle SES)

*"Um going round with my friends like chatting and things" *(Female, middle/high SES)

### Contexts of 'active play'

After the researcher had provided participants with a definition of active play, participants were asked to describe the contexts in which they engaged in active play, over the previous week. The reported contexts were divided into three themes according to: 1) What children do in their active play 2) Where they play and 3) Who they play with. These themes are discussed below.

### What children do in their active play

Participants were asked, "Over the past week, what did you do in your active play?" The activities children reported doing in their active play varied by gender. For the majority of boys, active play involved having a 'kick about' or riding bikes with friends:

*"Um I knock for my neighbours and we play football over the fields" *(Male, high SES)

*"Playing football over the parks" *(Male, low/middle SES)

*"I rode on my bike with my friends" *(Male, high SES)

*"Um like going around the streets on our bikes and stuff" *(Male, low/middle SES)

However, girls were less likely to describe a specific activity when discussing active play.

*"Um I go outside, run around" *(Female, low/middle SES)

*"...we live in a flat and there's like a little field of grass and we go down there and play*" (Female, low SES)

*"I play with my dogs outside" (*Female, high SES)

*"I play games with my brother" *(Female, middle/high SES)

### Where children engage in active play

Participants were then asked, "Over the past week, where did you play?" Female participants generally engaged in active play close to their homes, often in their own gardens:

*"I... go out in the garden ...that's basically all" *(Female, middle/high SES)

*"I went...in my front garden" *(Female, high SES)

*"I would normally go out in my back garden" *(Female, low SES)

*"Um in my garden and on the pavement outside my house" *(Female, low/middle SES)

In contrast, male participants appeared to have more independent mobility, and tended to play in neighbourhood green spaces or the streets:

*"I went to [name of park] just down my road, the new one which just opened" *(Male, low/middle SES)

*"I play on the community centre field" *(Male, high SES)

*"Streets" *(Male, middle/high SES)

*"Um me and my friends play where...literally anywhere, we pretty much ended up...so we went all round the streets everywhere really" *(Male, low SES)

### Who children engage in active play with

Finally, participants were asked the question "Over the past week, who did you play with?" with the follow-up probe, "School friends, neighbourhood friends, other friends, or family?" Many boys reported engaging in active play with their neighbourhood friends:

*"Well my best friend lives opposite me...and my other two friends don't live far so I just play with them" *(Male, high SES)

*"My next door neighbours and a friend who lives two doors down from me" *(Male, high SES)

*"Just people around the streets" *(Male, low SES)

*"Well it's my friend who lives just down the road" *(Male, middle/high SES)

However, girls were more likely to report engaging in active play with family members:

*"In school I've got some cousins so I normally play with them outside of school and some, family" *(Female, low SES)

*"I play with my brother" *(Female, low SES)

*"I play with my cousin, my grandparents and my um family at home" *(Female, high SES)

*"Um, I normally play um with my brother, my dog" *(Female, low/middle SES)

## Discussion

The data presented here indicate that many children perceive 'play' in terms of some form of physical activity, which in most cases is unstructured physical activity which takes place outdoors. This is typical of play in previous generations where children were reported to spend almost all of their free time outdoors [37] and is consistent with the current academic definition of 'active play' [25]. However, for some children, their perceptions of play correspond with indoor, more sedentary activities, which may reflect children's increasing use of indoor space at home as a venue for play [38,39]. Some of these sedentary play behaviours, such as playing computer games, could be targets for future physical activity interventions, either by reducing them or finding ways to increase the energy cost while performing them [40].

In terms of activities children engaged in as part of their active play, it was interesting to note that male participants were more likely to provide details of specific physical activities than females. This suggests that whilst having a 'kick about' in a field or cycling around the streets are reported as common leisure-time activities for boys, there may not be an equivalent unstructured physical activity for girls. This corresponds with a recent study which found 10-11 year old UK girls to view other aspects of life, such as socialising, to perhaps be more important than engaging in physical activity in their free time [41]. Thus, among girls, it may be particularly important to foster social networks in order to encourage active play [42]. In terms of the location of children's active play, male participants were more likely to report playing in neighbourhood green spaces than female participants. This finding supports a UK study with 10-14 year olds, which reported that playing fields and recreation grounds were labelled as 'boy spaces' but that equivalent 'girl spaces' were not identified [43]. It also supports recent research which demonstrated that boys may obtain more of their physical activity in neighbourhood green spaces than girls [44].

Girls were more likely to report that their active play involved playing in gardens with family members. As boys tended to report playing further afield with friends from their local neighbourhood, this may suggest that girls spend more time in environments where adults are in close proximity than boys. However, given that this issue was not directly discussed in the focus groups, it is an area that requires further research. It has previously been suggested that environments that promote greater independent mobility in children may increase their overall physical activity levels [45-47]. Therefore, finding ways to optimise girls' independent active play may be an important aim of future research [42] as this will encourage the additional health and social benefits that this form of physical activity provides for them. One approach would be examining why parents have become more anxious about letting their daughters play outdoors in recent times [48].

It is interesting to note that purpose-built playgrounds were rarely chosen as a destination for active play. This corresponds with previous research which suggested that, for children, less 'managed' spaces are more appealing [49] and that adult-designed playgrounds are currently unsuccessful in meeting children's needs or expectations in relation to play [50]. The findings also support those of a recent study of playgrounds in Spain, which found play equipment to be mainly suitable for younger children and that older children instead craved space to run around, kick balls or ride bicycles [51]. Collectively these findings raise questions about the UK Labour Government's £235 million investment in local play areas [52], which were designed to provide safe and accessible play opportunities for 8-13 year olds. Our data suggests that a formal evaluation of the effect of these programmes may be required, as it could be that the programmes are not having the intended effect and that the provision of open spaces is more preferable to purpose-built playgrounds amongst children of this age group.

### Limitations

Several important limitations of this study must be noted. Although the sample used in this study was reasonably large and socio-economically diverse, it is difficult to generalise the perceptions of our study sample to 10-11 year olds from the wider UK population. Secondly, questions relating to the contexts of children's play referred to experiences over the previous week, and thus may be subject to seasonal bias and participants' ability to recall this information. It is also important to recognize that as the data were collected in schools, only children attending schools on data collection days were able to participate. This means that the data presented here may not represent the play patterns of children who are frequently absent from school.

Although using qualitative methods that engage children directly provided valuable insight into their thoughts and feelings [53], it raised some methodological concerns. For example, it could be argued that the group setting can perpetuate conformity and the repetition of similar ideas [54]. However, the importance of providing honest, individual answers was stressed to participants and our findings illustrated that they were able to express different opinions. The research took place in a school setting, and this may have created a 'power difference' between the researcher and participants. However, although the focus group exercise bore some resemblance to everyday school activities, the researcher made an effort to create an environment that was non-threatening, confidential and stimulated the freedom to talk openly among participants. Finally, a broad approach was taken in this study in order to identify the key issues around modern children's play. Further work will be needed to provide greater detail on some of the emergent themes, such as the type of activities girls commonly engage in during play and the motivations behind children's choice of activities, location of activities, and who they engage in activities with.

## Conclusions

The data presented in this study suggest that 'play' is a broad and complex term for 10-11 year olds, and is interpreted as including both physically active as well as more sedentary behaviours. Contemporary children *do *engage in active play consistent with that of previous generations and value both the physical and social benefits it provides. However, whereas boys frequently report 'having a kick about' or riding bikes as their preferred forms of active play, girls are less likely to report an equivalent specific physical activity. Additionally, boys appear to have greater independent mobility in their active play than girls. Finally, boys are more likely to report playing with neighbourhood friends but girls are more often restricted to family-supervised play. Collectively, these findings suggest that promoting active play in children's leisure time may increase the physical activity of today's children, but that such strategies may need to be tailored according to gender. Further research is needed to establish the factors which support and constrain children's active play in order to develop environments that increase opportunities to engage in these behaviours.

## Competing interests

The authors declare that they have no competing interests.

## Authors' contributions

The study was designed by RB, RJ and KF. Analysis was performed by RB. The first draft of the paper was written by RB and all authors provided critical input and revisions. All authors read and approved the final manuscript.
